# Sumatriptan inhibits synaptic transmission in the rat midbrain periaqueductal grey

**DOI:** 10.1186/1744-8069-4-54

**Published:** 2008-11-11

**Authors:** Hyo-Jin Jeong, David Chenu, Emma E Johnson, Mark Connor, Christopher W Vaughan

**Affiliations:** 1 Pain Management Research Institute, Kolling Institute of Medical Research, Northern Clinical School, The University of Sydney at Royal North Shore Hospital, NSW 2065, Australia

## Abstract

**Background:**

There is evidence to suggest that the midbrain periaqueductal grey (PAG) has a role in migraine and the actions of the anti-migraine drug sumatriptan. In the present study we examined the serotonergic modulation of GABAergic and glutamatergic synaptic transmission in rat midbrain PAG slices in vitro.

**Results:**

Serotonin (5-hydroxytriptamine, 5-HT, IC_50 _= 142 nM) and the selective serotonin reuptake inhibitor fluoxetine (30 μM) produced a reduction in the amplitude of GABA_A_-mediated evoked inhibitory postsynaptic currents (IPSCs) in all PAG neurons which was associated with an increase in the paired-pulse ratio of evoked IPSCs. Real time PCR revealed that 5-HT1A, 5-HT1B, 5-HT1D and 5-HT1F receptor mRNA was present in the PAG. The 5-HT1A, 5-HT1B and 5-HT1D receptor agonists 8-OH-DPAT (3 μM), CP93129 (3 μM) and L694247 (3 μM), but not the 5-HT1F receptor agonist LY344864 (1 – 3 μM) inhibited evoked IPSCs. The 5-HT (1 μM) induced inhibition of evoked IPSCs was abolished by the 5-HT1B antagonist NAS181 (10 μM), but not by the 5-HT1A and 5-HT1D antagonists WAY100135 (3 μM) and BRL15572 (10 μM). Sumatriptan also inhibited evoked IPSCs with an IC_50 _of 261 nM, and reduced the rate, but not the amplitude of spontaneous miniature IPSCs. The sumatriptan (1 μM) induced inhibition of evoked IPSCs was abolished by NAS181 (10 μM) and BRL15572 (10 μM), together, but not separately. 5-HT (10 μM) and sumatriptan (3 μM) also reduced the amplitude of non-NMDA mediated evoked excitatory postsynaptic currents (EPSCs) in all PAG neurons tested.

**Conclusion:**

These results indicate that sumatriptan inhibits GABAergic and glutamatergic synaptic transmission within the PAG via a 5-HT1B/D receptor mediated reduction in the probability of neurotransmitter release from nerve terminals. These actions overlap those of other analgesics, such as opioids, and provide a mechanism by which centrally acting 5-HT1B and 5-HT1D ligands might lead to novel anti-migraine pharmacotherapies.

## Background

Migraine is an episodic disorder involving headache and other sensory disturbances, such as sensitivity to light and sound which can be accompanied by nausea, vomiting and an episodic visual disturbance or aura. Sumatriptan and other more recently developed triptans are 5-HT1B, 5-HT1D and 5-HT1F receptor agonists and have efficacy in the treatment of migraine [1-3]. The peripheral actions of triptans are thought to be mediated by intracranial blood vessel constriction and inhibition of CGRP release from afferent nerve terminals. Pain sensitive intracranial structures, such as the dura mater and superior sagittal sinus, project centrally to the medullary trigeminal nucleus [4-6]. Systemic administration and local microiontophoretic application of triptans inhibits activation of neurons in the trigeminal nucleus following intracranial vascular stimulation and this is likely to be mediated via 5-HT1B and 5-HT1D receptors [7-11]. Cellular and anatomical studies suggest that the triptan induced inhibition of afferent transmission into the trigeminal nucleus is mediated largely by 5-HT1D receptors located on the central terminals of trigeminal primary afferent fibres [11-13].

There is evidence from imaging studies and clinical reports that higher structures within the nervous system, such as the midbrain PAG, may be involved in the generation of migraine [14-17]. These findings are of particular interest due to the pivotal role of the PAG in integrating an animal's somatomotor, autonomic and behavioural responses to threat, stress and pain [18]. Furthermore, analgesic drugs such as opioids produce analgesia from within the PAG via a descending pathway which projects via the rostral ventromedial medulla to the spinal cord and medullary dorsal horn [19]. The PAG contains a dense plexus of serotonergic nerve terminals [20], and varying levels of 5-HT1A, 5-HT1B, 5-HT1D and 5-HT1F receptor mRNA and protein has been found in different species [21-25]. Electrical stimulation and microinjection of naratriptan into the PAG inhibits trigeminal neuronal responses to dural stimulation [26-28].

Functional and cellular studies have suggested that opioids produce analgesia from within the PAG by reducing GABAergic inhibition of output neurons which form part of an endogenous analgesic pathway [19]. It has been demonstrated that activation of presynaptic 5-HT1A receptors inhibits GABAergic synaptic transmission in dissociated PAG neurons [29], however, the role of 5-HT1B and 5-HT1D receptors in modulating GABAergic transmission within the PAG is unknown. In the present study, we examined the role of 5-HT1A, 5-HT1B, 5-HT1D and 5-HT1F receptor subtypes in the serotonergic modulation of synaptic transmission in midbrain PAG slices and whether these effects were mimicked by sumatriptan.

## Methods

Experiments were carried out on male and female Sprague-Dawley rats (16 – 28 days old) using a protocol approved by the Royal North Shore Hospital Animal Care and Ethics Committee. Animals were anaesthetised with isoflurane, decapitated and the brain rapidly removed and placed into ice-cold artificial cerebrospinal fluid (ACSF) of composition (mM): NaCl 126, KCl 2.5, NaH_2_PO_4 _1.4, MgCl_2 _1.2, CaCl_2 _2.4, glucose 11, NaHCO_3 _25, equilibrated with 95% O_2 _and 5% CO_2_. Coronal midbrain slices containing PAG were then cut (300 μm) using a vibratome (VT1000S, Leica Microsystems, Nussbloch, Germany), in ice-cold ACSF, as described previously [30].

### Electrophysiology

For electrophysiology experiments the slices were maintained at 34°C in a submerged chamber containing ACSF. The slices were then individually transferred to a recording chamber and superfused continuously (1.6 – 1.8 ml.min^-1^) with ACSF (34°C). PAG neurons were visualized using infra-red Dodt-tube optics on an upright microscope (Olympus BX50, Olympus, Sydney). Whole-cell voltage clamp recordings (holding potential -65 mV) were made using an Axopatch 200B (Molecular Devices, Sunnyvale, USA), with an internal solution of composition (mM): CsCl 140, HEPES 10, EGTA 0.2, MgCl_2 _1, MgATP 2, NaGTP 0.3 (pH 7.3, osmolarity 280 – 285 mOsm.l^-1^). Series resistance (< 20 MΩ) was compensated by 80% and continuously monitored during experiments.

In one series of experiments, electrically evoked synaptic currents were elicited in neurons every 12 s (stimuli: 1 – 50 V, 20 – 400 μs) via unipolar glass stimulating electrodes which were filled with ACSF and placed 40 – 200 μm from the recording electrode. Electrical stimuli were elicited either singly, or as paired stimuli of identical strength with an inter-stimulus interval of 70 ms for paired pulse experiments. In these experiments, GABA_A_-receptor mediated IPSCs were obtained in the presence of the non-NMDA receptor antagonist CNQX (5 μM) and the glycine receptor antagonist strychnine (5 μM); or non-NMDA mediated EPSCs were obtained in the presence of picrotoxin (100 μM) and strychnine (5 μM). In the second series of experiments, spontaneous miniature IPSCs were obtained in the presence of CNQX (5 μM), strychnine (5 μM) and tetrodotoxin (TTX, 300 nM).

### Analysis

IPSCs and EPSCs were filtered (2, 5 kHz low-pass filter) and sampled (5, 10 kHz) for on-line and later off-line analysis (Axograph X, Axograph Scientific Software, Sydney, Australia). Evoked IPSC and EPSC amplitude was measured relative to a 2 ms baseline period prior to the stimulus artefact of each evoked synaptic current. Miniature IPSCs were automatically detected with a sliding template algorithm, then manually checked offline. The threshold for miniature IPSC detection was adjusted to between 4.5 – 5.5 standard deviations above baseline noise, such that false positives and negatives accounted for less than 5% of the detected events. Plots of detected event rate versus time and cumulative probability distributions of event amplitudes and inter-event intervals were constructed. For concentration response analysis, a maximum of 2 – 3 concentrations of 5-HT, or sumatriptan which differed by at least 10 fold was applied to each neuron. The mean value at each concentration was calculated across all neurons and a concentration response curve was fit using a logistic function (Prism, Graphpad Software, San Diego, USA). Statistical comparisons between two groups were made using Student's paired t-tests, and those between more than two groups were made using a one-way analysis of variance followed by post-hoc comparisons using Dunnett's correction for multiple comparisons (Prism). Differences were considered significant when p < 0.05. All pooled data were expressed as means ± SEM. Stimulus artefacts have been partially blanked in figures of evoked IPSCs and EPSCs for clarity.

### Drugs

BRL15572 hydrochloride, CP93129, fluoxetine hydrochloride, L694247, LY344864, NAS-181, (2R)-(+)-8-Hydroxy-2-(di-n-propylamino)tetralin hydrobromide (8-OH-DPAT), SR95531 hydrobromide and (S)-WAY100135 dihydrochloride were obtained from Tocris Cookson (Bristol, UK); QX-314 bromide and TTX from Alomone Laboratories (Jerusalem, Israel); 6-cyano-7-nitroquinoxaline-2,3-dione disodium (CNQX), 5-hydroxytryptamine hydrochloride (5-HT), and strychnine hydrochloride from Sigma (Sydney, Australia). Sumatriptan succinate (12 mg.ml^-1 ^in saline, Imigran, Glaxo Smith Kline) was purchased over the counter. Stock solutions of all drugs were prepared in distilled water, or dimethyl sulfoxide, then diluted (1:1,000 – 1:10,000) to working concentrations using ACSF and applied by superfusion.

### PCR experiments

For RT-PCR experiments 500 μm thick rat brain slices were cut as described above and the PAG dissected using a fine needle. The medial dorsal raphe nucleus was excised from each slice. Total RNA was isolated using Tri Reagent (Sigma, Australia) as per manufacturer's instructions. In brief, RNA was extracted with chloroform in a series of centrifugation and suspension steps. The RNA-containing aqueous phase was precipitated in isopropanol and the pellet washed with 75% ethanol before re-suspending in MilliQ water. RNA quantity was determined with a ND-1000 spectrophotometer (NanoDrop Technologies Inc., USA) and quality assessed on a 1.2% formaldehyde gel. Total RNA (1 μg) was subject to reverse transcription using random hexamer primers (Fermentas International, Canada). The RNA/primer mixture was activated at 70°C for 5 minutes before the addition of 20 μl MasterMix (0.25 μl rRNasin (Promega, USA), 0.40 μl 100 mM dNTPs, 4 μl 5× Reaction buffer and 0.25 μl BioScript (all Bioline Australia Pty Ltd) in diethylpyrocarbonate treated H_2_O). The reactions were incubated for 60 minutes at 40°C before quenching for 10 minutes at 70°C. cDNA was stored at 4°C until further use. Expression of 5-HT receptors was assessed by polymerase chain reaction (RT-PCR) on a Mastercycler (Eppendorf AG, Germany). Each PCR amplification contained 1 μl cDNA, 12.5 μl 2× ImmoMix (Bioline Australia) and 0.75 μl forward/reverse primers for gene of interest (Table 1, Invitrogen Australia) supplemented with MilliQ H_2_O to a total reaction volume of 25 μl. Thermal cycling conditions were as follows: initial denaturation at 95°C for 10 minutes, followed by 40 cycles of 95°C for 20 s, 55°C for 20s and 72°C for 20s. A negative template control containing 1 μl MilliQ H_2_O was included for each set of primers. To confirm product presence and amplicon size, RT-PCR products were separated on 1.5% agarose gel and stained with Gel Red (Biotium, Hayward, California).

**Table 1:** The sequence of the primers used to amplify 5-HT1 receptor mRNA from PAG tissue.

Receptor	Product size (bp)	Primers
5-HT1A	113	5'-TCTGTTGCTGGGTACTCTCA-3'
		5'-AGGAGCCGATGAGATAGTTG-3'
5-HT1B	151	5'-ATGGAGGAGCAGGGTATTCA-3'
		5'-AAGCAACCAGCAGGACTTTC-3'
5-HT1D	171	5'-ATCTCAGATTTCTTACACCATCTACTC-3'
		5'-CGCAGAGCCCGTGATAAG-3'
5-HT1F	108	5'-ACCACCCAGCCAACTATTTA-3'
		5'-TAATCCAACTCTCGCTCACA-3'

For each receptor the forward and reverse primers are shown, respectively. bp is base pairs.

## Results

### Exogenous and endogenous 5-HT inhibit GABAergic synaptic transmission

**Figure 1:**
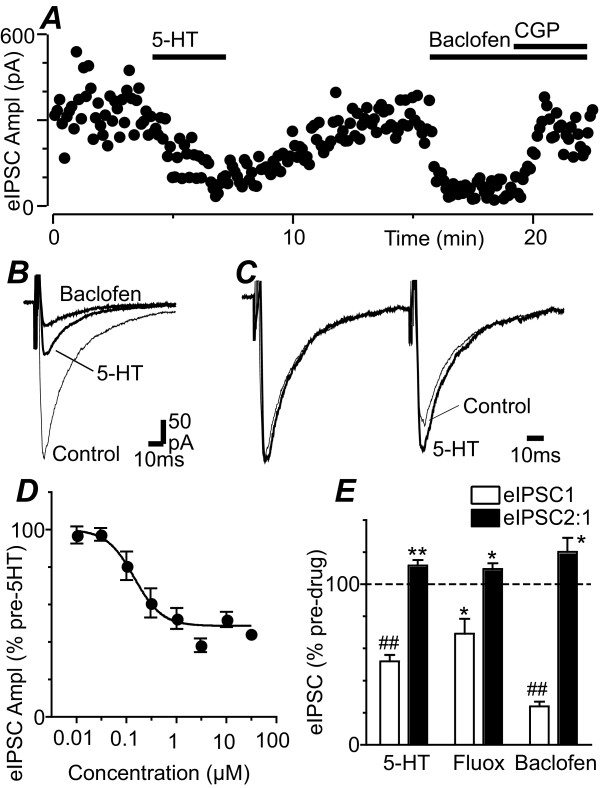
**5-HT inhibits evoked IPSCs in PAG neurons**. (A) Time course of evoked IPSC amplitude (eIPSC Ampl) during application of 5-HT (10 μM), baclofen (10 μM) and CGP55845 (CGP, 1 μM). (B) Averaged evoked IPSCs before (control) and during application of 5-HT and baclofen. (C) Averaged evoked IPSCs in response to identical paired stimuli (inter-stimulus interval = 70 ms) for the traces in B, with IPSC_1 _normalized to demonstrate relative facilitation of IPSC_2 _during superfusion of 5-HT. (D) Concentration-response curve of the reduction in evoked IPSC (eIPSC) amplitude produced by 5-HT, expressed as a percentage of the pre-5-HT level, with a logistic function fitted to determine the IC_50_. (E) Bar chart showing the amplitude of the first evoked IPSC (eIPSC1) and the ratio of evoked IPSC_2_/IPSC_1 _(eIPSC2:1) in the presence of 5-HT (10 μM), fluoxetine (30 μM) and baclofen (10 μM), expressed as a percentage of the pre-drug level. In (D) *, ** and ## denote p < 0.05, 0.01 and 0.0001. Traces in (A – C) are from the same neuron.

In the presence of CNQX (5 μM) and strychnine (5 μM), local electrical stimulation evoked IPSCs in PAG neurons which were abolished by tetrodotoxin (300 nM, n = 3) and by the GABA_A _antagonist SR95531 (10 μM, n = 3). When 5-hydroxytriptamine (5-HT, 10 μM) was superfused onto midbrain slices, it produced a reduction in the amplitude of evoked IPSCs in all PAG neurons tested which reversed following washout (Figure 1a, b, e, p < 0.0001, n = 25). The inhibition of evoked IPSCs produced by 5-HT (10 μM) was similar for neurons within the ventrolateral, lateral and dorsal/dorsolateral PAG columns (p = 0.9, n = 8, 10, 7). The 5-HT induced inhibition of evoked IPSCs was concentration dependent, with an IC_50 _of 142 nM (90% confidence interval = 75 – 273 nM) and a Hill slope of 1.5 ± 0.5 (Figure 1d). Superfusion of the selective serotonin reuptake inhibitor fluoxetine (30 μM) also produced a significant reduction in the amplitude of evoked IPSCs which reversed upon washout (Figure 1e, p = 0.02, n = 7). Subsequent superfusion of the GABA_B _agonist baclofen (10 μM) produced a reduction in the amplitude of evoked IPSCs which was reversed by addition of the GABA_B _antagonist CGP55845 (10 μM) (Figure 1a, b, e, p < 0.0001, n = 14). 5-HT, fluoxetine and baclofen had no effect on membrane current, or the conductance of the neurons at -65 mV.

In some of these recordings paired evoked IPSCs were elicited by paired stimuli of equal strength (inter-stimulus interval = 70 ms). Before drug application the ratio of evoked IPSC_2_/IPSC_1 _was 1.09 ± 0.04, with both paired pulse depression and facilitation being observed (range = 0.63 – 1.95, n = 37). 5-HT (10 μM), fluoxetine (30 μM) and baclofen (10 μM) all produced a significant increase in the ratio of evoked IPSC_2_/IPSC_1 _(Figure 1c, e, p = 0.003, 0.04, 0.03, n = 21, 7, 14).

### 5-HT1A, 5-HT1B and 5-HT1D receptor activation inhibits GABAergic synaptic transmission

**Figure 2:**
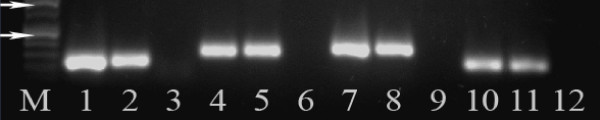
**mRNA for 5-HT1A, B, D and F receptors is expressed in rat PAG**. A representative agarose gel illustrating amplified fragments of mRNA for each of four 5-HT1 receptor subtypes in PAG slices of a male and a female rat. The lane marked M is the molecular weight ladder, the arrows indicate 200 D (lower) and 500 D (upper), respectively. The first lane for each receptor is from a male rat, the second from a female, the third is the no-template control for reagent contamination. 5-HT1A is Lanes 1–3, 5-HT1B is Lanes 4–6, 5-HT1D is Lanes 7–9, 5-HT1F is lanes 10–12. The primers used to amplify the mRNA are listed in Table 1.

We first examined which of the 5-HT1 receptors are present within the PAG by real time PCR. Amplification of total PAG cDNA with primers designed for four 5-HT1 receptor subtypes resulted in single bands of the expected size (Figure 2, n = 4 for each). Both male and female rats expressed mRNA for 5-HT1A, B, D and F receptors. Each of the primer pairs we used amplified a single mRNA species of the appropriate size (Table 1).

**Figure 3:**
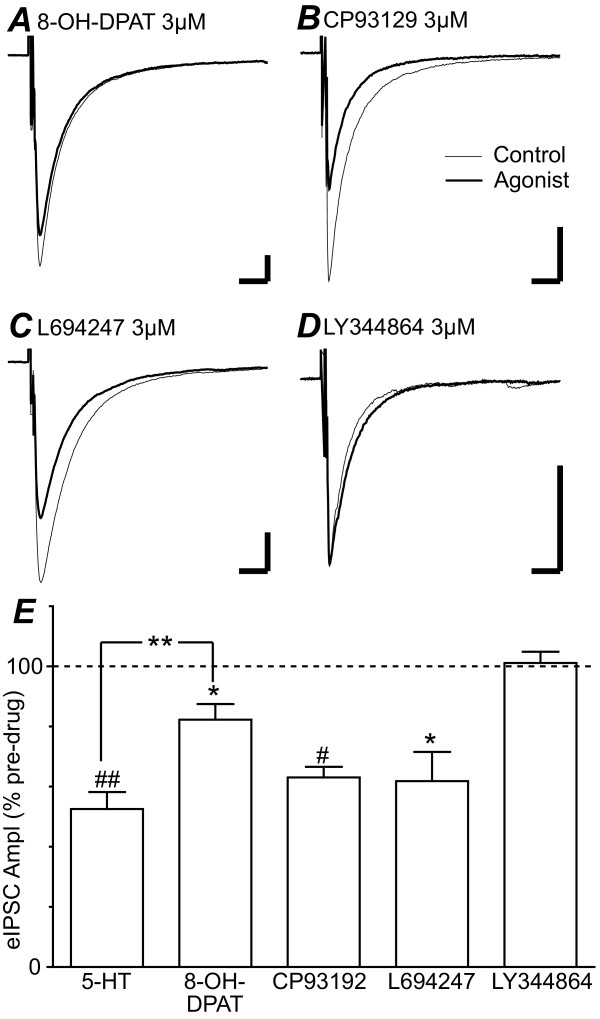
**5-HT1A, 5-HT1B and 5-HT1D receptor activation inhibits evoked IPSCs in PAG neurons**. Averaged evoked IPSCs are shown prior to (control) and during (agonist) addition of (A) the 5-HT1A agonist 8-OH-DPAT (3 μM), (B) the 5-HT1B agonist CP93129 (3 μM), (C) the 5-HT1D agonist L694247 (3 μM) and (D) the 5-HT1F agonist LY344864 (3 μM). (E) Bar chart showing the percentage inhibition of evoked IPSC amplitude (eIPSC Ampl) produced by 5-HT (1 μM), 8-OH-DPAT (3 μM), CP93129 (3 μM) L694247 (3 μM) and LY344864 (1 – 3 μM) expressed as a percentage of the pre-drug level. In (E), *, **, # and ## denote p < 0.05, 0.01, 0.001 and 0.0001. Traces in (A – D) are from different neurons, bars are 200 pA and 10 ms.

We then determined which 5-HT1 receptor subtypes modulated GABAergic synaptic transmission by testing the effect of a range of 5-HT1 selective agonists. The 5-HT1A selective agonist 8-OH-DPAT (3 μM) produced a reduction in the amplitude of evoked IPSCs (Figure 3a, e, p = 0.03, n = 5) which was less than that produced by 5-HT (1 μM) (Figure 3e, p < 0.01). The 5-HT1B selective agonist CP93129 (3 μM) produced a reduction in the amplitude of evoked IPSCs (Figure 3b, e, p = 0.0004, n = 5). The 5-HT1D selective agonist L694247 (3 μM) also produced a reduction in the amplitude of evoked IPSCs (Figure 3c, e, p = 0.02, n = 5). The reduction in evoked IPSC amplitude produced by CP93129 and L694247 was not significantly different to that produced by 5-HT (1 μM) (Figure 3e, p > 0.05). In contrast, the 5-HT1F selective agonist LY344864 (1 – 3 μM) did not produce a significant change in the amplitude of evoked IPSCs (Figure 3d, e, p = 0.8, n = 7).

**Figure 4:**
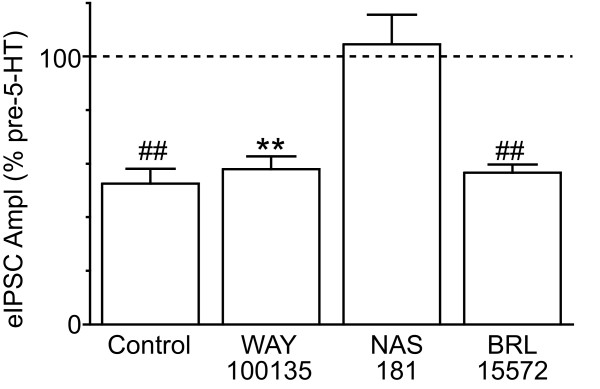
**5-HT inhibition of evoked IPSCs is largely mediated by 5-HT1B receptors**. Bar chart displaying the mean reduction in evoked IPSC amplitude produced by 5-HT (1 μM) in neurons which were either not pre-incubated in any antagonist (control), or were pre-incubated in the 5-HT1A antagonist WAY100135 (3 μM), the 5-HT1B antagonist NAS181 (10 μM), or the 5-HT1D antagonist BRL15572 (10 μM). Evoked IPSC amplitude is expressed as a percentage of the pre-5-HT level. **, ## denote p < 0.01, 0.0001.

We next examined the contribution of 5-HT1A, 5-HT1B and 5-HT1D receptors to the 5-HT induced inhibition by pre-incubating slices in 5-HT1A, B, or D antagonists for 10 minutes prior to the addition of 5-HT (1 μM). In the presence of the 5-HT1A antagonist WAY100135 (3 μM), 5-HT (1 μM) produced a significant reduction in evoked IPSC amplitude (Figure 4, p = 0.001, n = 5). Similarly, in the presence of the 5-HT1D antagonist BRL15572 (10 μM), 5-HT (1 μM) produced a significant reduction in evoked IPSC amplitude (Figure 4, p = 0.0006, n = 5). The inhibition produced by 5-HT in the presence of WAY100135, or BRL15572 was not significantly different from that produced in their absence (Figure 4, p > 0.05). By contrast, 5-HT (1 μM) did not produce a significant reduction in evoked IPSC amplitude in the presence of the 5-HT1B antagonist NAS181 (10 μM) (Figure 4, p = 0.7, n = 7).

### Sumatriptan inhibits evoked GABAergic synaptic currents via a presynaptic mechanism

**Figure 5:**
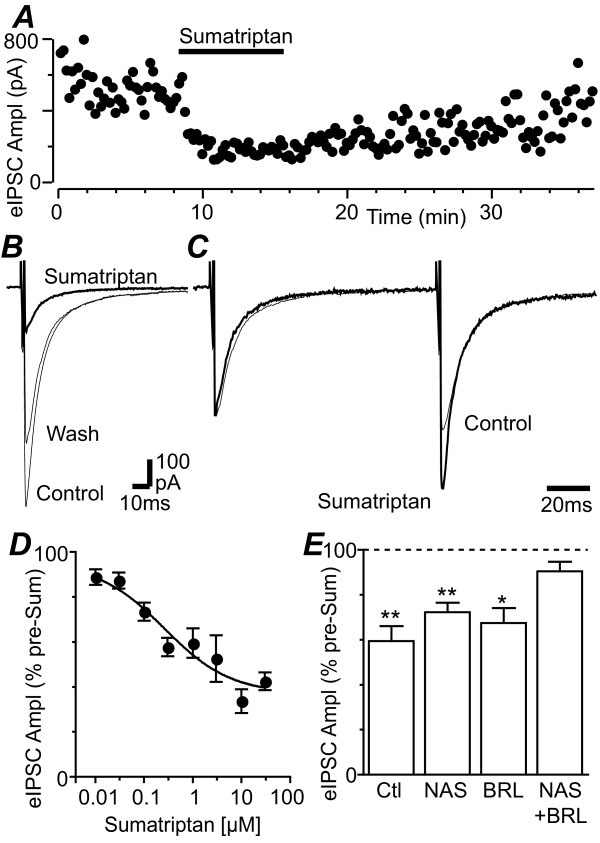
**Sumatriptan inhibits evoked IPSCs in PAG neurons**. (A) Time course of evoked IPSC amplitude (eIPSC Ampl) during application of sumatriptan (3 μM). (B) Averaged evoked IPSCs before (control), during application of sumatriptan (3 μM) and following washout (wash). (C) Averaged evoked IPSCs in response to identical paired stimuli (IPSC1-2 interval = 70 ms) for the traces in B, with IPSC_1 _normalized to demonstrate relative facilitation of IPSC_2 _during superfusion of sumatriptan (3 μM). (D) Concentration-response curve of the reduction in evoked IPSC amplitude produced by sumatriptan, expressed as a percentage of the pre-sumatriptan level, with a logistic function fitted to determine the IC_50_. (E) Bar chart showing the evoked IPSC amplitude in the presence of sumatriptan (1 μM), expressed as a percentage of the pre- sumatriptan level, in neurons which received no prior treatment (control) and those which were pre-incubated in BRL15572 (10 μM) and/or NAS181 (10 μM). In (E) *, ** denote p < 0.05, 0.01. Traces in (A – C) are from the same neuron.

When sumatriptan (1 – 10 μM) was superfused onto midbrain slices, it produced a reduction in the amplitude of evoked IPSCs in all PAG neurons tested (Figure 5a, b, p < 0.001, n = 16). The inhibition of evoked IPSCs produced by sumatriptan was concentration dependent, with an IC_50 _of 261 nM (90% confidence interval = 42 – 1,610 nM) and a Hill slope of 0.64 ± 0.22 (Figure 5d). The sumatriptan induced inhibition only partially reversed after a lengthy washout period at concentrations of 1 μM and above (Figure 5a), as observed by others [13,31]. Sumatriptan (1 – 10 μM) also increased the ratio of evoked IPSC_2_/IPSC_1 _from 1.26 ± 0.1 to 1.37 ± 0.1 (Figure 5c, p = 0.02, n = 16). Sumatriptan had no effect on membrane current, or conductance of neurons at -65 mV.

We also examined the contribution of 5-HT1B and 5-HT1D receptors to the inhibition produced by sumatriptan by pre-incubating slices in 5-HT1B and 5-HT1D antagonists. Sumatriptan (1 μM) produced a significant reduction in evoked IPSC amplitude in the presence of NAS181 (10 μM, p = 0.003, n = 5), or BRL15572 (10 μM, p = 0.02, n = 4) which was not significantly different to that produced in their absence (Figure 5e, p > 0.05). By contrast, sumatriptan (1 μM) did not produce a significant reduction in evoked IPSC amplitude in the presence of both NAS181 (10 μM) and BRL15572 (10 μM) (Figure 5e, p = 0.1, n = 5).

**Figure 6:**
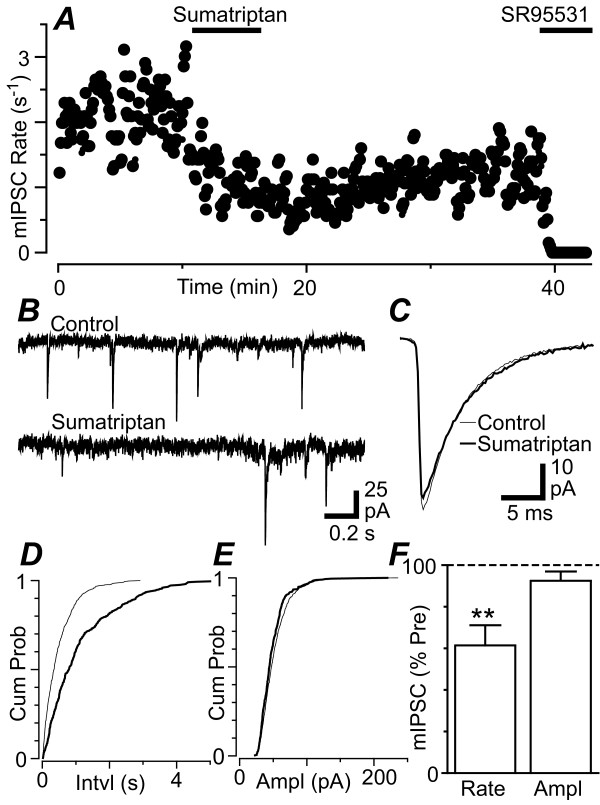
**Sumatriptan decreases the rate, but not the amplitude of miniature IPSCs**. (A) Time course of miniature IPSC (mIPSC) rate during superfusion of sumatriptan (3 μM) and SR95531 (10 μM). (B) Raw current traces of miniature IPSCs before (control) and during superfusion of sumatriptan (3 μM). (C) Averaged traces of miniature IPSCs before and during superfusion of sumatriptan (3 μM). Cumulative distribution plots of miniature IPSC (D) inter-event interval and (E) amplitude, before and during sumatriptan (3 μM). (F) Bar chart of the mean rate and amplitude of miniature IPSCs in the presence of sumatriptan (3 μM), expressed as a percentage of the pre-sumatriptan level, ** denotes p < 0.01. Traces in (A – E) are from the same neuron.

In the presence of TTX (300 nM), CNQX (5 μM) and strychnine (5 μM) spontaneous miniature IPSCs were observed which were abolished by SR95531 (10 μM, n = 4) (Figure 6a). Sumatriptan (3 μM) produced a reduction in the rate of miniature IPSCs in the majority of PAG neurons tested (Figure 6a, b, f, p = 0.007, n = 7). The reduction in miniature IPSC rate produced by sumatriptan only partially reversed after a lengthy washout period (Figure 6a), as observed for evoked IPSCs. The sumatriptan induced reduction in miniature IPSC rate was associated with a rightward shift in the cumulative probability distribution of miniature IPSC inter-event intervals (Figure 6d). By contrast, sumatriptan did not produce a change in the amplitude (p = 0.2) or kinetics of miniature IPSCs, or in the cumulative probability distributions of mIPSC amplitudes (Figure 6c, e, f).

### Serotonin and sumatriptan inhibit evoked glutamatergic synaptic currents

**Figure 7:**
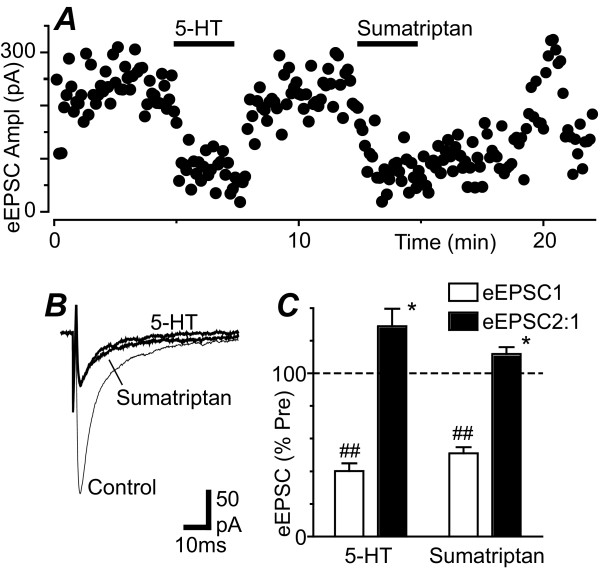
**5-HT inhibits evoked EPSCs in PAG neurons**. (A) Time course of evoked EPSC amplitude (eEPSC Ampl) during application of 5-HT (10 μM) and sumatriptan (3 μM). (B) Averaged evoked EPSCs before (control) and during application of 5-HT and sumatriptan. (C) Bar chart showing the amplitude of the first evoked EPSC (eEPSC1) and the ratio of evoked EPSC_2_/EPSC_1 _(eEPSC2:1) in the presence of 5-HT (10 μM) and sumatriptan (3 μM), expressed as a percentage of the pre-drug level. In (C) * and ## denote p < 0.05 and 0.0001. Traces in (A – B) are from the same neuron.

In the presence of picrotoxin (100 μM) and strychnine (5 μM), local electrical stimulation evoked EPSCs in PAG neurons. Superfusion of 5-HT (10 μM) produced a significant reduction in the amplitude of evoked EPSCs which reversed following washout (Figure 7a–c, p < 0.0001, n = 6). Sumatriptan (3 μM) produced a significant reduction in the amplitude of evoked EPSCs which slowly, and only partially reversed upon washout (Figure 7a–c, p < 0.0001, n = 6). The inhibition of evoked EPSCs produced by both 5-HT (10 μM) and sumatriptan (3 μM) was associated with a significant increase in the ratio of evoked EPSC_2_/EPSC_1 _(Figure 7c, p = 0.04, n = 6).

## Discussion

The present study has demonstrated that the anti-migraine drug sumatriptan inhibits GABAergic and glutamatergic synaptic transmission within the PAG via a 5-HT1B and 5-HT1D receptor mediated decrease in neurotransmitter release from presynaptic nerve terminals. These findings suggest that, in addition to inhibition of afferent transmission into the medullary dorsal horn, 5-HT1B and 5-HT1D ligands might also have a central role in the control of pain by activating, or disinhibiting descending analgesic pathways.

A number of observations suggested that sumatriptan, and exogenous and endogenous 5-HT inhibit GABAergic and glutamatergic synaptic transmission via a presynaptic reduction in neurotransmitter release, similar to that previously reported for opioids and cannabinoids within the PAG [30,32,33]. In the present study, 5-HT and sumatriptan reduced the amplitude of evoked GABA_A _mediated IPSCs and non-NMDA mediated EPSCs which was associated with an increase in the paired-pulse ratio of evoked IPSCs and EPSCs. The 5-HT and sumatriptan inhibition of evoked IPSCs was concentration dependent and was mimicked by the selective serotonin reuptake inhibitor fluoxetine. In addition, sumatriptan produced a reduction in the frequency, but not the amplitude of spontaneous miniature IPSCs, as observed for miniature EPSCs in the spinal trigeminal nucleus [13,31]. These observations are consistent with prior studies which have observed serotonergic presynaptic inhibition of GABAergic synaptic transmission within the PAG, spinal trigeminal nucleus and other brain regions [13,29,34-38]. These findings are also consistent with the observations that sumatriptan inhibits GABAergic synaptic transmission in the ventral tegmental area and presynaptically inhibits glutamatergic synaptic transmission in the spinal trigeminal nucleus [13,31,39].

The present study has demonstrated that functional 5-HT1A, 5-HT1B and 5-HT1D receptors are present on GABAergic nerve terminals within the PAG. It was found that GABAergic synaptic transmission was inhibited by the selective 5-HT1A, 5-HT1B and 5-HT1D agonists 8-OH-DPAT, CP93129 and L694247, but not by the 5-HT1F agonist LY344864. This is consistent with a previous study which reported that 5-HT1A receptor activation presynaptically inhibits GABAergic synaptic transmission within the PAG [29]. While the cellular actions of 5-HT1B, 5-HT1D and 5-HT1F ligands have not been examined in the PAG previously, the current findings are consistent with studies demonstrating 5-HT1B receptor induced inhibition of GABAergic synaptic transmission in the adjacent dorsal raphe nucleus [35] and other brain regions [34,36-40]. To our knowledge there are no other studies which have reported 5-HT1D receptor mediated presynaptic inhibition of GABAergic synaptic transmission, although, 5-HT1D inhibition of glutamatergic and serotonergic transmission has been observed within the spinal trigeminal nucleus and cortex [13,41,42]. These findings are consistent with the detection of mRNA for each of 5-HT1A, 5-HT1B and 5-HT1D receptors in the present study in both male and female rats, although the present study was not designed to examine quantitative gender related differences. While previous experiments in rat using autoradiography and *in situ *hybridization have demonstrated the presence of 5-HT1A and 5-HT1B receptors in PAG, 5-HT1D receptor binding or mRNA was undetectable [21,22,25]. While the lack of 5-HT1F mediated inhibition is not consistent with the detection of mRNA for 5-HT1F receptors in the present study, very low levels of 5-HT1F mRNA have been reported in guinea pig [25].

The present findings suggest that 5-HT1B and 5-HT1D receptors play a predominant role in the presynaptic modulation of GABAergic synaptic transmission by 5-HT and sumatriptan within the PAG. It was observed that the inhibition of GABAergic synaptic transmission produced by 5-HT was abolished by the 5-HT1B antagonist NAS181, but not by the 5-HT1A and 5-HT1D antagonists WAY100135 and BRL15572, while that produced by sumatriptan was only abolished in the combined presence of NAS181 and BRL15572. The lack of a significant role for 5-HT1A receptors in the actions of 5-HT, or sumatriptan is consistent with the finding that the inhibition of GABAergic synaptic transmission produced by 8-OH-DPAT was less than that produced by 5-HT, CP93129 and L694247. It is also consistent with prior studies which have reported that 5-HT1A levels within the PAG are relatively low compared to the adjacent dorsal raphe nucleus, and that 5-HT1A and 5-HT1B receptors are largely expressed within postsynaptic and presynaptic elements within the midbrain, respectively [43,44]. In this regard, it has previously been shown that 5-HT1A receptor activation produces postsynaptic inhibition within the PAG [45]. The difference between the effects of 5-HT1B and 5-HT1D antagonists on the 5-HT and sumatriptan induced inhibition might be related to differences in their potency at these receptor subtypes [46], or to redundancy in receptor-effector coupling such as that observed for 5-HT1B and 5-HT2 receptors in the inferior olive [47].

Functional studies suggest that opioids and cannabinoids can reduce ascending nociceptive transmission arising from cutaneous structures by activating a descending pathway which projects from the PAG to the spinal cord dorsal horn via the rostral ventromedial medulla [19]. Similarly, electrical stimulation and microinjection of naratriptan into the ventrolateral PAG selectively inhibits the responses of trigeminal neurons to stimulation of the dura mater and superior sagittal sinus, indicating that triptans activate a descending analgesic pathway to the medullary dorsal horn [27,28]. Activation of the PAG-spinal cord pathway by analgesics such as opioids and cannabinoids is thought to be mediated by a reduction in the GABAergic inhibition of PAG and rostral ventromedial medulla output neurons, a process known as disinhibition. At the cellular level, disinhibition within the PAG is partly mediated by presynaptic inhibition of neurotransmitter release from the terminals of GABAergic neurons. The present findings have demonstrated that sumatriptan, acting via 5-HT1B and 5-HT1D receptors, produces presynaptic disinhibition, similar to that previously observed for μ-opioid, cannabinoid CB_1 _and 5-HT1A receptor agonists [29,30,33]. In the present study it was also observed that sumatriptan inhibited glutamatergic synaptic transmission which would be expected to functionally oppose GABAergic disinhibition. It might be noted, however, that opioids and cannabinoids, both of which produce analgesia from within the PAG, also inhibit glutamatergic synaptic transmission within this brain structure [30,33]. It is also possible that 5-HT and sumatriptan modulate neuronal excitability by direct 5-HT1B, D and F receptor mediated postsynaptic actions, as previously demonstrated for μ-opioid and 5-HT1A receptor agonists [45,48,49]. Neuronal excitability within the PAG is likely to be regulated in a complex manner by presynaptic GABAergic and glutamatergic inputs and by direct postsynaptic actions, as previously demonstrated for μ-opioids [50], and the net effect of serotonergic agents on neuronal activity within the PAG remains to be determined.

Prior studies have shown that the ventrolateral PAG differs from other, more dorsal PAG subregions in that it is activated by pain from deep, rather than superficial structures [18] and by trigeminovascular stimulation [51,52]. The present observation that 5-HT and sumatriptan inhibited synaptic transmission throughout the dorsolateral, lateral and ventrolateral PAG suggests that triptans might have a more generalised role in pain modulation. It has been reported that, in addition to acute trigeminovascular pain, triptans have some efficacy in animal models of inflammation and trigeminal, but not sciatic nerve related neuropathic pain [53,54]. The variable efficacy of triptans in these pain models might be related to differences in their effects on trigeminal versus spinal nociceptive inputs [27,55], and/or to differences in their ability to cross the blood brain barrier [see [53,56]]. While it is unclear whether systemically administered sumatriptan accesses central structures such as the PAG at therapeutic levels, triptans with better brain penetration might provide an alternative approach. In this regard, the observed 5-HT1D receptor involvement in PAG disinhibition is particularly interesting because it has a more restricted distribution with the central nervous system compared to 5-HT1B receptors [23-25] and could potentially provide a selective pharmacological tool in the treatment of migraine and related pain states [see [57]].

## Conclusion

The present cellular findings indicate that the anti-migraine drug sumatriptan activates, or disinhibits descending analgesic pathways from within the PAG via activation of 5-HT1B and 5-HT1D receptors. This disinhibition is at least partly mediated by a decrease in neurotransmitter release from GABAergic nerve terminals and overlaps the mechanism of action other analgesic agents within this brain structure. The widespread inhibition of GABAergic and glutamatergic synaptic transmission by sumatriptan within the PAG observed in the present study suggests that centrally acting 5-HT1B/D agonists might have a more generalised role in the control of pain and the integrated somatomotor, autonomic and behavioural functions of this brain structure.

## List of abbreviations

ACSF: artificial cerebrospinal fluid; CNQX: 6-cyano-7-nitroquinoxaline-2,3-dione disodium; EPSC: excitatory postsynaptic current; 5-HT: 5-hydroxytriptamine; IPSC: inhibitory postsynaptic current; 8-OH-DPAT: (2R)-(+)-8-Hydroxy-2-(di-n-propylamino)tetralin hydrobromide; PAG: periaqueductal grey.

## Competing interests

The authors declare that they have no competing interests.

## Authors' contributions

DC, HJJ and CWV performed the patch clamp experiments and analysed electrophysiological data. EEJ and MC performed the PCR experiments and analysed the PCR data. All authors contributed to making the figures and writing the manuscript, and they all read and approved the final manuscript. The study was conceived and the experiments were designed by CWV and MC.
